# Editorial: State-of-the-art rational nanodesign: from screening to theranostics and from bench to clinic

**DOI:** 10.3389/fphar.2023.1210185

**Published:** 2023-05-17

**Authors:** Alicia Fernandez-Fernandez, Romila Manchanda, João Manuel Cunha Rodrigues, Yuan Tang

**Affiliations:** ^1^ College of Health Care Sciences, Nova Southeastern University, Fort Lauderdale, FL, United States; ^2^ Institute for Quantitative Health Science and Engineering, Michigan State University, East Lansing, MI, United States; ^3^ Centro de Química da Madeira, Universidade da Madeira, Funchal, Portugal; ^4^ Bioengineering Department, University of Toledo, Toledo, OH, United States

**Keywords:** nanotechnology, nanomaterials, clinical translation, nanodesign, theranostics, nanomedicine

The field of nanotherapeutics has grown immensely in the past few decades, including both disease-specific nano-based approaches to diagnosis and therapy, as well as molecule-oriented strategies which are not necessarily limited to a specific disease, but rather aim to enhance therapeutic delivery of a particular active principle or family of drugs with subsequent applications to multiple pathological processes (Anjum et al., 2021; Mitchell et al., 2021). Nanoformulations can improve biocompatibility and have several advantages, such as adaptability and amenability to targeted design with a variety of potential applications including drug delivery, diagnostic techniques, and multifunctional platforms that allow for screening, diagnosis, therapy, and theranostic approaches (Loo et al., 2022; Zhu et al., 2022; Thapa et al., 2023). This Research Topic highlights the current state of the art in nano-engineered formulations in cancer, generic joint osteoarthritis, and temporomandibular joint osteoarthritis, including summaries of existing approaches as well as bench-to-clinic translational challenges. The issue also covers fabrication trends such as biogenic synthesis, and includes a manuscript focused on gingerols, a specific family of active agents with potential for therapeutic use in multiple diseases, and their nano formulations.

In “*Lipid-engineered nanotherapeutics for cancer management*,” Fernandez-Fernandez et al. present a scoping review of current applications of lipid-based nanosystems in the management of cancer. Cancer nanotherapeutics attempts to overcome some of the challenges of traditional cancer interventions such as chemotherapy and radiotherapy. Nanodesigns can enhance bioavailability, improve biodistribution, provide targeting opportunities, enhance tumor site uptake, and tailor treatments to the cancer microenvironment, as well as provide multifunctional approaches that streamline care. The authors specifically discuss the applications of liposomal therapeutics, lipidic nanoemulsions, solid lipid nanoparticles, nanostructured lipidic carriers, lipid-polymer nanohybrids, and supramolecular nanolipidic structures. The manuscript also analyzes the successes and challenges of bench-to-clinic transition, including an overview of existing patents, clinical trials, and marketed formulations; along with barriers such as bioenvironment variability, scalability, quality control, regulatory uncertainty, and long-term effects.

The article “*Therapeutic potential of nanotechnology-based approaches in osteoarthritis*” by Xiao et al. reviews the potential benefits of using nanotechnology-based approaches in the treatment of osteoarthritis (OA). OA is a degenerative joint disease that affects millions of people worldwide and is characterized by the breakdown of joint cartilage. In the article, the authors discuss various nanotechnology-based approaches that have been explored in preclinical and clinical studies, including nanoparticles for drug delivery, nanofibers for tissue engineering, and nanosensors for disease monitoring. The authors highlight the potential of these approaches in addressing current challenges in OA treatment, such as specifically targeting drugs to the affected joint, increasing treatment efficacy, and extending drug release through sustained-release formulations. The article also discusses the potential of nanotechnology-based approaches to explore underlying mechanisms of OA, such as inflammation and oxidative stress. Overall, the article suggests that nanotechnology-based approaches could lead to more effective and targeted OA therapies in the future.

In “*Drug delivery systems for treatment of temporomandibular joint (TMJ) osteoarthritis*”, Huang et al. describe yet another application for nanoengineered vehicles in optimizing medical care, in this case for individuals with TMJ degenerative disease. In TMJ osteoarthritis, mechanisms such as chondrocyte apoptosis, extracellular matrix degeneration, joint inflammation, and subchondral bone remodeling result in pain and functional limitation. Traditional anti-inflammatory treatment approaches do not address the multifactorial pathological changes that occur, and they are also limited by their bioavailability profiles. Novel drug delivery systems, particularly those involving lipid-engineered nano vehicles, can improve targeted delivery and bioavailability of biomolecules that address not only inflammation but also other aspects of osteoarthritis, such as promoting the repair of cartilaginous damage. The authors describe various drug delivery systems, including intra-articular and transdermal approaches, and within them, different reported designs to maximize therapeutic potential. Finally, the authors discuss the outlook of this research area, including targeting, long-term effect, and toxicity considerations.

The synthesis of metallic nanoparticles using natural sources, such as nontoxic plant extracts (biogenic synthesis), has attracted the interest of academia and companies worldwide due to the reported advantages over the use of hazardous chemical reagents commonly used in preparing nanoparticles. In the mini-review “*Bibliometric analysis on exploitation of biogenic gold and silver nanoparticles in breast, ovarian and cervical cancer therapy*”, Bhandari et al. analyze bibliometric data from 2010 to 2022 on the biogenic synthesis of gold and silver nanoparticles for treating cancer, particularly breast, ovarian, and cervical cancer. The review also highlights the main biological and technological challenges that limit their use in clinics. The authors discuss the importance of using this sustainable approach in nanoparticle preparation, and they emphasize that in order to overcome existing bottlenecks that limit clinical trial success and subsequent market deployment, it will be essential to complete further studies on the molecular mechanisms of action of biogenic nanoparticles, combined with molecular modeling.

Finally, in “*Immunomodulatory and anti-inflammatory therapeutic potential of gingerols and their nanoformulations*”, Yücel et al. discuss the various biomedical applications of gingerols, compounds present in ginger, which have various pharmacological effects including anti-inflammatory, antinociceptive, antioxidant, antimicrobial, anti-cancer, anti-hyperglycemic, and anti-arteriosclerotic, among others. Gingerols are also immunomodulators, and they influence cell adhesion, migration, invasion, and apoptosis, making them potentially useful in various diseases. Unfortunately, they are also poorly soluble, which results in low bioavailability with low absorption and rapid metabolism after oral administration. The authors discuss how novel nanoformulations can improve the delivery and bioavailability of gingerols in multiple applications; and how nano-design can enhance stability, achieve sustained release, increase efficacy, and reduce toxicity. Some of the formulations that are discussed include nanovesicles, proliposomes, nanostructured lipid carriers, microemulsions, and nanoparticles, among others, with the potential for applications in a variety of diseases.

Nanotechnology shows excellent potential for improving patient care and quality of life through approaches that maximize therapeutic precision while minimizing undesirable effects, allowing researchers and clinicians to collaborate in designing treatment strategies that are disease-specific and ultimately tailored to each patient. A consistent, unified regulatory environment needs to be further developed to successfully support this vision. We hope that this Research Topic gives the readers a small window into a growing, promising, and very attractive field.
